# Stable reinforcement learning via temporal competition between LTP and LTD traces

**DOI:** 10.1186/1471-2202-15-S1-O12

**Published:** 2014-07-21

**Authors:** Marco A Huertas, Sarah Schwettmann, Alfredo Kirkwood, Harel Shouval

**Affiliations:** 1 University of Texas Medical School, Dep. Neurobiology and Anatomy, Houston, TX 77030, USA; 2 Rice University, Dep. Computational and Applied Mathematics, Houston, TX 77005, USA; 3 Johns Hopkins University, Mind/Brain Institute, Baltimore, MD, 21218, USA

## 

Neuronal systems that are involved in reinforcement learning must solve the temporal credit assignment problem, i.e., how is a stimulus associated with a reward that is delayed in time? Theoretical studies [1-3] have postulated that neural activity underlying learning ‘tags’ synapses with an ‘eligibility trace’, and that the subsequent arrival of a reward converts the eligibility traces into actual modification of synaptic efficacies. While eligibility traces provide one simple solution to the temporal credit assignment problem, they alone do not constitute a stable learning rule because there is no other mechanism indicating when learning should cease. In order to attain stability, rules involving eligibility traces often assume that once the association is learned, further learning is prevented via an inhibition of the reward stimulus [1,3,4].

Although synaptic plasticity is responsible for reinforcement learning in the brain, theories of reinforcement learning are generally abstract and involve neither neurons nor synapses. Furthermore, biophysical theories of synaptic plasticity typically model unsupervised learning and ignore the contribution of reinforcement. Here we describe a biophysically based theory of reinforcement-modulated synaptic plasticity and postulate the existence of two eligibility traces with different temporal profiles: one corresponding to the induction of LTP, and the other to the induction of LTD. The traces have different kinetics and their difference in magnitude at the time of reward determines if synaptic modification will correspond to LTP or LTD. Due to the difference in their decay rates, the LTP and LTD traces can exhibit temporal competition at the reward time and thus provides a mechanism for stable reinforcement learning without the need to inhibit reward. We test this novel reinforcement-learning rule on an experimentally motivated model of a recurrent cortical network [5], and compare the model results to experimental results at both the cellular and circuit levels. We further suggest that these eligibility traces are implemented via kinases and phosphatases, thus accounting for results at both the cellular and system levels.
